# *N*-(2-Carb­oxy­eth­yl)-2,5-dide­oxy-2,5-imino-d-mannonic acid [(3*R*,4*R*,5*R*)-1-(2-carb­oxy­eth­yl)-3,4-dihy­droxy-5-hy­droxy­methyl-l-proline]

**DOI:** 10.1107/S1600536812037488

**Published:** 2012-09-05

**Authors:** David S. Edgeley, R. Fernando Martínez, Sarah F. Jenkinson, Robert J. Nash, George W. J. Fleet, Amber L. Thompson

**Affiliations:** a Department of Chemical Crystallography, Chemistry Research Laboratory, University of Oxford, Oxford OX1 3TA, England; b Department of Organic Chemistry, Chemistry Research Laboratory, University of Oxford, Oxford OX1 3TA, England; c Phytoquest Limited, IBERS, Plas Gogerddan, Aberystwyth, Ceredigion SY23 3EB, Wales

## Abstract

The absolute stereochemistry of the title compound, C_9_H_15_NO_7_, was determined from the use of d-glucuronolactone as the starting material. The compound crystallizes as the zwitterion. The five-membered ring adopts an envelope conformation with the –CH_2_OH-substituted C atom forming the flap. An intramolecular N—H⋯O hydrogen-bond occurs. In the crystal, the compound exists as a three-dimensional O—H⋯O intermolecular hydrogen-bonded network with each mol­ecule acting as a donor and acceptor for four hydrogen bonds.

## Related literature

For related literature on naturally occurring imino­sugars, see: Asano *et al.* (2000 ▶); Watson *et al.* (2001 ▶); Nash *et al.* (1991 ▶); Welter *et al.* (1976 ▶); Manning *et al.* (1985 ▶); Pereira *et al.* (1991 ▶). For the synthesis of the diacid, see: Best *et al.* (2010 ▶); Martínez *et al.* (2012 ▶). For the extinction correction, see: Larson (1970 ▶). For hydrogen-atom refinement, see: Cooper *et al.* (2010 ▶). For the temperature controller, see: Cosier & Glazer (1986 ▶). For the Chebychev polynomial used in the weighting scheme, see: Prince (1982 ▶); Watkin (1994 ▶).
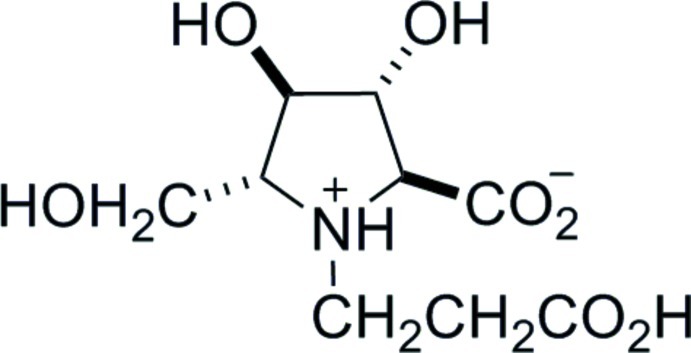


## Experimental

### 

#### Crystal data

C_9_H_15_NO_7_
*M* *_r_* = 249.22
Orthorhombic, 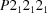

*a* = 8.5242 (1) Å
*b* = 8.5707 (1) Å
*c* = 14.3585 (3) Å
*V* = 1049.01 (3) Å^3^
*Z* = 4
Mo *K*α radiation
μ = 0.14 mm^−1^
*T* = 190 K
0.32 × 0.30 × 0.11 mm

#### Data collection

Nonius KappaCCD diffractometer
Absorption correction: multi-scan (*DENZO*/*SCALEPACK*; Otwinowski & Minor, 1997 ▶) *T*_min_ = 0.93, *T*_max_ = 0.99
18435 measured reflections
1385 independent reflections
1321 reflections with *I* > 2σ(*I*)
*R*_int_ = 0.009

#### Refinement

*R*[*F* ^2^ > 2σ(*F* ^2^)] = 0.024
*wR*(*F* ^2^) = 0.062
*S* = 0.93
1385 reflections
155 parameters
H-atom parameters constrained
Δρ_max_ = 0.21 e Å^−3^
Δρ_min_ = −0.14 e Å^−3^

### 

Data collection: *COLLECT* (Nonius, 2001 ▶); cell refinement: *DENZO*/*SCALEPACK* (Otwinowski & Minor, 1997 ▶); data reduction: *DENZO*/*SCALEPACK*; program(s) used to solve structure: *Superflip* (Palatinus & Chapuis, 2007 ▶); program(s) used to refine structure: *CRYSTALS* (Betteridge *et al.*, 2003 ▶); molecular graphics: *CAMERON* (Watkin *et al.*, 1996 ▶); software used to prepare material for publication: *CRYSTALS*.

## Supplementary Material

Crystal structure: contains datablock(s) global, I. DOI: 10.1107/S1600536812037488/lh5524sup1.cif

Structure factors: contains datablock(s) I. DOI: 10.1107/S1600536812037488/lh5524Isup2.hkl

Additional supplementary materials:  crystallographic information; 3D view; checkCIF report

## Figures and Tables

**Table 1:** Hydrogen-bond geometry (Å, °)

*D*—H⋯*A*	*D*—H	H⋯*A*	*D*⋯*A*	*D*—H⋯*A*
O17—H171⋯O15^i^	0.84	2.19	2.887 (2)	141
O14—H141⋯O10^ii^	0.86	1.78	2.618 (2)	166
O1—H11⋯O10^iii^	0.82	1.95	2.753 (2)	165
O4—H41⋯O9^i^	0.82	1.93	2.720 (2)	163
N6—H61⋯O15	0.90	2.15	2.768 (2)	125

Symmetry codes: (i) 
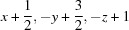
; (ii) 
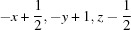
; (iii) 
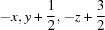
.

**Fig. 1.**
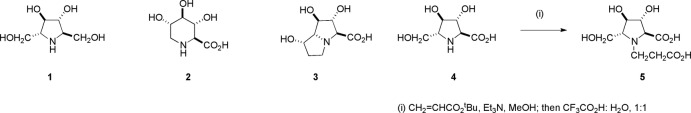
Synthetic Scheme.

**Fig. 2.**
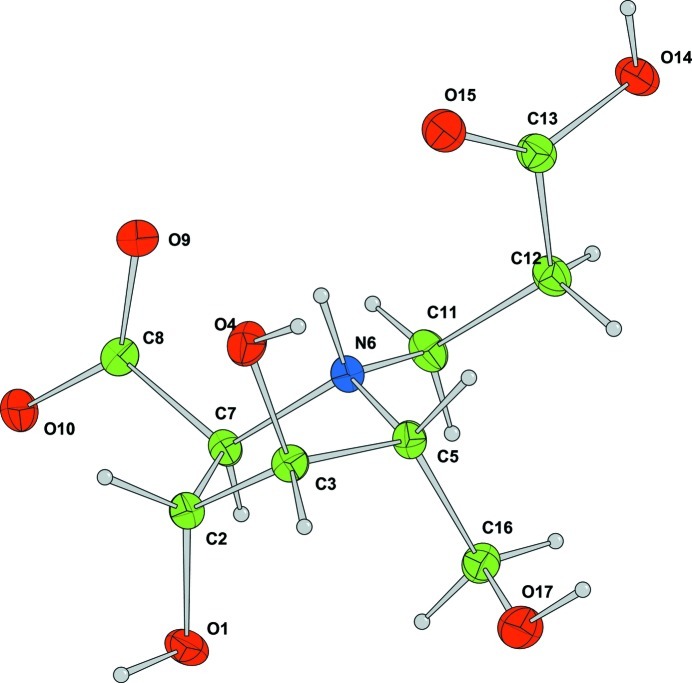
The title compound with displacement ellipsoids drawn at the 50% probability level. H atoms are shown as spheres of arbitrary radius.

**Fig. 3.**
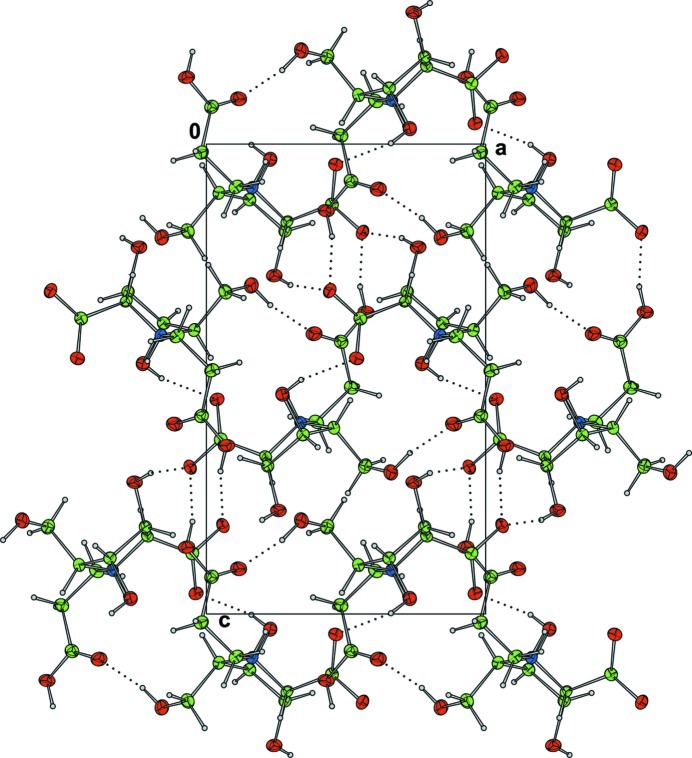
Packing diagram of the title compound projected along the b-axis. Hydrogen bonds are shown as dotted lines.

## supplementary crystallographic information

### Comment

More than 250 iminosugars, sugar mimics in which the ring oxygen in a pyranose or furanose is replaced by nitrogen to form polyhydroxylated piperidines and pyrrolidines, have been isolated from plants (Asano *et al.*, 2000; Watson *et al.*, 2001). DMDP 1 (Fig. 1), originally isolated from *Derris eliptica* (Welter *et al.*, 1976), but the most widely occurring iminosugar, is even found in potatoes (Nash *et al.*, 1991). In contrast, BR1 2 from *Baphia racemosa* (Manning *et al.*, 1985) and 7a-epialexaflorine 3 from *Alexa grandiflora* (Pereira *et al.*, 1991) are among the very few corresponding sugar amino acids (SAA) found in nature. From examination of crude extracts of plants, it is clear that other SAA are natural products. As part of a program to make authentic samples of such SAA to identify them in crude plant extracts, the SAA corresponding to DMDP 4 (Best *et al.*, 2010) was converted to the diacid 5 by initial reaction with *tert*-butyl acrylate in methanol in the presence of triethylamine followed by treatment with aqueous trifluoroacetic acid (Martínez, 2012). The structure of 5 was unequivocally determined by X-ray crystallographic analysis; the absolute configuration was determined by the use of *d*-glucuronolactone as the starting material for the synthesis.

The five ring adopts an envelope conformation with C5 out of the plane (Fig. 2). The compound exisits as a three-dimensional O—H···O intermolecular hydrogen-bonded network with each molecule acting as a donor and acceptor for four hydrogen bonds (Fig. 3).

### Experimental

The synthetic procedure is described in the comment section and illustrated in Fig. 1. The title compound was recrystallized from water: [α]_D_^25^ -6.7 (*c* 0.75 in H_2_O); m.p. 523 K (decomposed).

### Refinement

In the absence of significant anomalous scattering, Friedel pairs were merged and the absolute configuration was assigned from the starting material.

The H atoms were all located in a difference map, but those attached to carbon atoms were repositioned geometrically. The H atoms were initially refined with soft restraints on the bond lengths and angles to regularize their geometry (C—H in the range 0.93–0.98, O—H = 0.82 Å) and *U*ĩso~(H) (in the range 1.2–1.5 times *U*~eq~ of the parent atom), after which the positions were refined with riding constraints (Cooper *et al.*, 2010).

### Crystal data

**Table:**

C_9_H_15_NO_7_	*F*(000) = 528
*M**_r_* = 249.22	*D*_x_ = 1.578 Mg m^−^^3^
Orthorhombic, *P*2_1_2_1_2_1_	Mo *K*α radiation, λ = 0.71073 Å
Hall symbol: P 2ac 2ab	Cell parameters from 1401 reflections
*a* = 8.5242 (1) Å	θ = 5–27°
*b* = 8.5707 (1) Å	µ = 0.14 mm^−^^1^
*c* = 14.3585 (3) Å	*T* = 190 K
*V* = 1049.01 (3) Å^3^	Plate, colourless
*Z* = 4	0.32 × 0.30 × 0.11 mm

### Data collection

**Table:**

Nonius KappaCCD diffractometer	1321 reflections with *I* > 2σ(*I*)
Graphite monochromator	*R*_int_ = 0.009
ω scans	θ_max_ = 27.5°, θ_min_ = 5.3°
Absorption correction: multi-scan (*DENZO*/*SCALEPACK*; Otwinowski & Minor, 1997)	*h* = −11→11
*T*_min_ = 0.93, *T*_max_ = 0.99	*k* = −11→11
18435 measured reflections	*l* = −18→18
1385 independent reflections	

### Refinement

**Table:**

Refinement on *F*^2^	Hydrogen site location: difference Fourier map
Least-squares matrix: full	H-atom parameters constrained
*R*[*F*^2^ > 2σ(*F*^2^)] = 0.024	Method, part 1, Chebychev polynomial, (Watkin, 1994; *P*rince, 1982) [*w*eight] = 1.0/[A_0_*T_0_(x) + A_1_*T_1_(x) ··· + A_n-1_]*T_n-1_(x)] where A_i_ are the Chebychev coefficients listed belo*w* and x = *F* /*F*max Method = Robust Weighting (*P*rince, 1982) W = [*w*eight] * [1-(delta*F*/6*sigma*F*)^2^]^2^ A_i_ are: 24.6 39.9 23.9 10.2 2.48
*wR*(*F*^2^) = 0.062	(Δ/σ)_max_ = 0.0004353
*S* = 0.93	Δρ_max_ = 0.21 e Å^−^^3^
1385 reflections	Δρ_min_ = −0.14 e Å^−^^3^
155 parameters	Extinction correction: Larson (1970), Equation 22
0 restraints	Extinction coefficient: 190 (20)
Primary atom site location: Other	

### Special details

**Table:**

Experimental. The crystal was placed in the cold stream of an Oxford Cryosystems open-flow nitrogen cryostat (Cosier & Glazer, 1986) with a nominal stability of 0.1 K.

### Fractional atomic coordinates and isotropic or equivalent isotropic displacement parameters (Å^2^)

**Table:**

	*x*	*y*	*z*	*U*_iso_*/*U*_eq_	
O1	0.25779 (14)	0.79237 (13)	0.78008 (7)	0.0203	
C2	0.21884 (17)	0.76687 (17)	0.68515 (10)	0.0156	
C3	0.34616 (17)	0.82784 (16)	0.61866 (10)	0.0153	
O4	0.27181 (12)	0.86546 (13)	0.53261 (7)	0.0199	
C5	0.45582 (16)	0.68918 (15)	0.60409 (9)	0.0148	
N6	0.33646 (14)	0.56002 (13)	0.59290 (8)	0.0141	
C7	0.21204 (16)	0.58889 (17)	0.66539 (10)	0.0144	
C8	0.05081 (18)	0.54093 (16)	0.62872 (10)	0.0155	
O9	0.03760 (13)	0.51412 (12)	0.54371 (7)	0.0196	
O10	−0.05866 (13)	0.53953 (13)	0.68791 (7)	0.0205	
C11	0.39382 (17)	0.39407 (16)	0.59147 (11)	0.0168	
C12	0.51896 (18)	0.36273 (17)	0.51838 (9)	0.0177	
C13	0.48212 (17)	0.41891 (17)	0.42111 (10)	0.0178	
O14	0.57147 (14)	0.35134 (13)	0.35801 (7)	0.0218	
O15	0.38504 (14)	0.51908 (13)	0.40387 (7)	0.0241	
C16	0.56756 (17)	0.66168 (17)	0.68540 (11)	0.0191	
O17	0.66018 (13)	0.79709 (15)	0.69892 (8)	0.0252	
H21	0.1206	0.8129	0.6680	0.0190*	
H31	0.3983	0.9179	0.6450	0.0177*	
H51	0.5150	0.6992	0.5452	0.0178*	
H71	0.2358	0.5326	0.7211	0.0155*	
H111	0.3007	0.3304	0.5802	0.0199*	
H112	0.4377	0.3698	0.6517	0.0210*	
H121	0.5390	0.2510	0.5152	0.0211*	
H122	0.6181	0.4132	0.5352	0.0221*	
H162	0.6327	0.5676	0.6725	0.0238*	
H161	0.5065	0.6450	0.7429	0.0230*	
H171	0.7288	0.8063	0.6573	0.0384*	
H141	0.5503	0.3854	0.3035	0.0342*	
H11	0.1995	0.8611	0.8000	0.0308*	
H41	0.3391	0.9087	0.5016	0.0308*	
H61	0.2882	0.5748	0.5379	0.0220*	

### Atomic displacement parameters (Å^2^)

**Table:**

	*U*^11^	*U*^22^	*U*^33^	*U*^12^	*U*^13^	*U*^23^
O1	0.0242 (5)	0.0209 (5)	0.0158 (5)	0.0028 (5)	0.0009 (4)	−0.0050 (4)
C2	0.0150 (6)	0.0164 (6)	0.0153 (6)	0.0016 (5)	0.0004 (5)	−0.0010 (5)
C3	0.0158 (6)	0.0143 (6)	0.0158 (6)	0.0003 (5)	−0.0004 (5)	0.0005 (5)
O4	0.0173 (5)	0.0230 (5)	0.0195 (5)	0.0015 (4)	0.0003 (4)	0.0065 (4)
C5	0.0133 (6)	0.0146 (6)	0.0166 (6)	−0.0001 (5)	0.0012 (5)	−0.0005 (5)
N6	0.0134 (5)	0.0142 (5)	0.0148 (5)	0.0008 (4)	0.0011 (5)	−0.0004 (4)
C7	0.0144 (6)	0.0148 (6)	0.0140 (6)	0.0014 (5)	0.0020 (5)	0.0002 (5)
C8	0.0161 (6)	0.0137 (6)	0.0167 (6)	−0.0004 (5)	−0.0008 (5)	0.0016 (5)
O9	0.0196 (5)	0.0233 (5)	0.0160 (5)	0.0003 (5)	−0.0016 (4)	−0.0015 (4)
O10	0.0154 (5)	0.0266 (5)	0.0196 (5)	−0.0028 (4)	0.0036 (4)	−0.0011 (4)
C11	0.0202 (7)	0.0124 (6)	0.0179 (6)	0.0024 (5)	0.0038 (6)	0.0012 (5)
C12	0.0198 (7)	0.0157 (6)	0.0176 (6)	0.0034 (6)	0.0020 (6)	−0.0005 (5)
C13	0.0184 (6)	0.0176 (6)	0.0174 (6)	−0.0005 (6)	0.0013 (6)	−0.0020 (5)
O14	0.0257 (6)	0.0246 (5)	0.0151 (5)	0.0056 (5)	0.0016 (4)	−0.0016 (4)
O15	0.0254 (5)	0.0277 (6)	0.0191 (5)	0.0085 (5)	−0.0010 (5)	0.0005 (4)
C16	0.0168 (6)	0.0195 (7)	0.0209 (7)	0.0018 (6)	−0.0022 (6)	−0.0029 (6)
O17	0.0189 (5)	0.0334 (6)	0.0232 (5)	−0.0089 (5)	0.0013 (5)	−0.0051 (5)

### Geometric parameters (Å, º)

**Table:**

O1—C2	1.4198 (17)	C7—H71	0.956
O1—H11	0.822	C8—O9	1.2471 (17)
C2—C3	1.537 (2)	C8—O10	1.2622 (18)
C2—C7	1.553 (2)	C11—C12	1.5204 (19)
C2—H21	0.958	C11—H111	0.976
C3—O4	1.4257 (16)	C11—H112	0.965
C3—C5	1.5263 (19)	C12—C13	1.5103 (19)
C3—H31	0.968	C12—H121	0.974
O4—H41	0.815	C12—H122	0.980
C5—N6	1.5121 (17)	C13—O14	1.3177 (18)
C5—C16	1.525 (2)	C13—O15	1.2178 (19)
C5—H51	0.989	O14—H141	0.855
N6—C7	1.5064 (17)	C16—O17	1.4171 (18)
N6—C11	1.5041 (17)	C16—H162	0.997
N6—H61	0.900	C16—H161	0.987
C7—C8	1.528 (2)	O17—H171	0.840
			
C2—O1—H11	107.6	C2—C7—H71	109.5
O1—C2—C3	112.26 (12)	N6—C7—H71	110.3
O1—C2—C7	109.60 (12)	C8—C7—H71	110.1
C3—C2—C7	104.29 (11)	C7—C8—O9	117.91 (13)
O1—C2—H21	112.8	C7—C8—O10	115.82 (12)
C3—C2—H21	108.5	O9—C8—O10	126.19 (15)
C7—C2—H21	109.0	N6—C11—C12	113.87 (12)
C2—C3—O4	107.53 (11)	N6—C11—H111	105.5
C2—C3—C5	104.65 (11)	C12—C11—H111	110.9
O4—C3—C5	109.24 (11)	N6—C11—H112	108.5
C2—C3—H31	110.6	C12—C11—H112	108.0
O4—C3—H31	111.2	H111—C11—H112	110.1
C5—C3—H31	113.2	C11—C12—C13	115.85 (12)
C3—O4—H41	105.3	C11—C12—H121	109.2
C3—C5—N6	99.93 (10)	C13—C12—H121	107.9
C3—C5—C16	113.45 (11)	C11—C12—H122	111.0
N6—C5—C16	112.86 (11)	C13—C12—H122	105.4
C3—C5—H51	111.2	H121—C12—H122	107.1
N6—C5—H51	108.4	C12—C13—O14	112.05 (12)
C16—C5—H51	110.5	C12—C13—O15	123.65 (13)
C5—N6—C7	106.26 (10)	O14—C13—O15	124.25 (14)
C5—N6—C11	118.36 (11)	C13—O14—H141	111.0
C7—N6—C11	113.18 (11)	C5—C16—O17	109.05 (12)
C5—N6—H61	107.3	C5—C16—H162	109.3
C7—N6—H61	105.2	O17—C16—H162	112.2
C11—N6—H61	105.7	C5—C16—H161	109.5
C2—C7—N6	105.15 (11)	O17—C16—H161	107.4
C2—C7—C8	111.15 (12)	H162—C16—H161	109.4
N6—C7—C8	110.54 (11)	C16—O17—H171	111.5

### Hydrogen-bond geometry (Å, º)

**Table:**

*D*—H···*A*	*D*—H	H···*A*	*D*···*A*	*D*—H···*A*
C5—H51···O4^i^	0.99	2.52	3.366 (2)	143 (1)
C7—H71···O17^ii^	0.96	2.49	3.352 (2)	151 (1)
C11—H112···O17^ii^	0.97	2.38	3.156 (2)	137 (1)
C12—H121···O9^iii^	0.97	2.43	3.354 (2)	160 (1)
C12—H122···O4^i^	0.98	2.50	3.257 (2)	134 (1)
C16—H161···O1	0.99	2.53	3.174 (2)	123
O17—H171···O15^i^	0.84	2.19	2.887 (2)	141 (1)
O14—H141···O10^iv^	0.86	1.78	2.618 (2)	166 (1)
O1—H11···O10^v^	0.82	1.95	2.753 (2)	165 (1)
O4—H41···O9^i^	0.82	1.93	2.720 (2)	163 (1)
N6—H61···O15	0.90	2.15	2.768 (2)	125

Symmetry codes: (i) *x*+1/2, −*y*+3/2, −*z*+1; (ii) −*x*+1, *y*−1/2, −*z*+3/2; (iii) *x*+1/2, −*y*+1/2, −*z*+1; (iv) −*x*+1/2, −*y*+1, *z*−1/2; (v) −*x*, *y*+1/2, −*z*+3/2.

## References

[bb1] Asano, N., Nash, R. J., Molyneux, R. J. & Fleet, G. W. J. (2000). *Tetrahedron Asymmetry*, **11**, 1645–1680.

[bb2] Best, D., Wang, C., Weymouth-Wilson, A. C., Clarkson, R. A., Wilson, F. X., Nash, R. J., Miyauchi, S., Kato, A. & Fleet, G. W. J. (2010). *Tetrahedron Asymmetry*, **21**, 311–319.

[bb3] Betteridge, P. W., Carruthers, J. R., Cooper, R. I., Prout, K. & Watkin, D. J. (2003). *J. Appl. Cryst.* **36**, 1487.

[bb4] Cooper, R. I., Thompson, A. L. & Watkin, D. J. (2010). *J. Appl. Cryst.* **43**, 1100–1107.

[bb5] Cosier, J. & Glazer, A. M. (1986). *J. Appl. Cryst.* **19**, 105–107.

[bb6] Larson, A. C. (1970). *Crystallographic Computing*, edited by F. R. Ahmed, S. R. Hall & C. P. Huber, pp. 291–294. Copenhagen: Munksgaard.

[bb7] Manning, K. S., Lynn, D. G., Shabanowitz, J., Fellows, L. E., Sin gh, M. & Schrire, B. D. (1985). *J. Chem. Soc. Chem. Commun.* pp. 127–129.

[bb8] Martínez, R. F., Jenkinson, S. F., Hollas, M., Ayres, B. J., Nash, R. J., Kato, A. & Fleet, G. W. J. (2012). In preparation.

[bb9] Nash, R. J., Watson, A. A., Winters, A. L., Fleet, G. W. J., Wormald, M. R., Dealler, S., Lees, E., Asano, N. & Kizu, H. (1991). *Spec. Publ. R. Soc. Chem.* **200**, 106–114.

[bb10] Nonius (2001). *COLLECT* Nonius BV, Delft, The Netherlands.

[bb11] Otwinowski, Z. & Minor, W. (1997). *Methods in Enzymology*, Vol. 276, *Macromolecular Crystallography*, Part A, edited by C. W. Carter Jr & R. M. Sweet, pp. 307–326. New York: Academic Press.

[bb12] Palatinus, L. & Chapuis, G. (2007). *J. Appl. Cryst.* **40**, 786–790.

[bb13] Pereira, A. C. de S., Kaplan, M. A. C., Maia, J. G. S., Gottlieb, O. R., Nash, R. J., Fleet, G., Pearce, L., Watkin, D. J. & Scofield, A. M. (1991). *Tetrahedron*, **47**, 5637–5640.

[bb14] Prince, E. (1982). In *Mathematical Techniques in Crystallography and Materials Science* New York: Springer-Verlag.

[bb15] Watkin, D. (1994). *Acta Cryst.* A**50**, 411–437.

[bb16] Watkin, D. J., Prout, C. K. & Pearce, L. J. (1996). *CAMERON* Chemical Crystallography Laboratory, Oxford, England.

[bb17] Watson, A. A., Fleet, G. W. J., Asano, N., Molyneux, R. J. & Nash, R. J. (2001). *Phytochemistry*, **56**, 265–295. 10.1016/s0031-9422(00)00451-9 11243453

[bb18] Welter, A., Jadot, J., Dardenne, G., Marlier, M. & Casimir, J. (1976). *Phytochemistry*, **15**, 747–749.

